# Stress, Dietary Patterns and Cardiovascular Disease: A Mini-Review

**DOI:** 10.3389/fnins.2019.01226

**Published:** 2019-11-12

**Authors:** Luis Pedro Morera, Georgina Noel Marchiori, Leonardo Adrián Medrano, María Daniela Defagó

**Affiliations:** ^1^Instituto de Organizaciones Saludables, Universidad Siglo 21, Córdoba, Argentina; ^2^Instituto de Investigaciones en Ciencias de la Salud (INICSA), CONICET, Universidad Nacional de Córdoba, Córdoba, Argentina; ^3^Escuela de Nutrición, Facultad de Ciencias Médicas, Universidad Nacional de Córdoba, Córdoba, Argentina; ^4^Department of Pyschology, Pontificia Universidad Católica Madre y Maestra, Santiago de los Caballeros, Dominican Republic

**Keywords:** stress, cardiovascular disease, dietary patterns, nutrition, microbiome

## Abstract

According to the World Health Organization, an unhealthy diet and insufficient physical activity are the leading global risks to health. Dietary behavior is a modifiable factor in cardiovascular disease (CVD) prevention. Furthermore, the fact that cardiovascular events and stress-related emotional disorders share a common epidemiology may indicate the existence of pathways linking these two diseases (Chauvet-Gelinier and Bonin, 2017). Psychosocial stress can lead to changes in dietary patterns (DP) and under chronic stress conditions, high caloric and hyperpalatable foods are preferred. The interplay between these two factors impacts on several biological pathways: for example, it can prime the hippocampus to produce a potentiated neuroinflammatory response, generating memory deficits; it can also affect gut microbiota composition, ultimately influencing behavior and brain health and creating a predisposition to the development of diseases such as obesity, CVD, diabetes and metabolic syndrome. Though both cognition and emotion can be heavily affected by caloric intake, diet composition and stress, the molecular pathways involved remain elusive (Spencer et al., 2017). In this review, we describe the interplay between stress and DP at a molecular level, and how these factors relate to brain health and mental fitness. Finally, we show how these findings could give rise to novel therapeutic targets for chronic diseases.

## Introduction

Cardiovascular disease (CVD), is the leading cause of mortality and disability worldwide. According to the Global Burden of Disease study, ischemic heart disease and stroke accounted for 25% of total deaths worldwide in 2013 (Rubinstein et al., 2010; GBD 2013 Mortality and Causes of Death Collaborators, 2015).

The current epidemic of CVD is largely explained by several modifiable risk factors associated with lifestyle, feasible to modify. In this sense, an unbalanced diet, excessive alcohol and tobacco consumption, hypercholesterolemia, diabetes mellitus, high blood pressure, visceral obesity, physical inactivity and psychosocial stress increase the risk of future CVD events and are responsible for an estimated 90% of the population-attributable risk fraction of ischemic heart disease and stroke worldwide (Schächinger et al., 2000; O’Donnell et al., 2010; Uthman et al., 2014; Karmali et al., 2017). Even more stress-related psychological disorders such as depression show a higher prevalence in patients with coronary artery disease or heart failure compared with the general population (Lane et al., 2002; Ruo et al., 2003; Rutledge et al., 2006).

**FIGURE 1:**
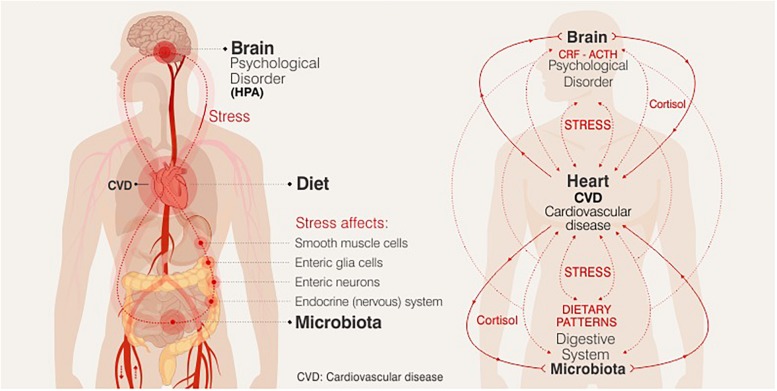
Relationship between nutritional changes and emotional state and brain health.

Chronic or acute exposure to stress (defined as an ongoing or anticipated threat to homeostasis or well-being) favors to the destabilization of the dynamic balance of the organism, and its response promotes the release of chemical mediators that affect the metabolic and behavioral state in humans. Stress-induced activation of the neuroendocrine hypothalamic-pituitary-adrenal axis (HPA), upon exposure to a stressor cortisol is expected to exert widespread metabolic effects, which is mostly necessary to maintain or restore homeostasis. Stress, perhaps, is the trigger in the cascade of neuroendocrine effects that drive the development of the visceral distribution of adipose tissue, resistance to insulin and the consequent hyperinsulinemia, thus leading to the accumulation of cardiovascular risk factors (Serrano Ríos, 2005). Since glucocorticoids favor increased adiposity, mainly, abdominal fat, they can lead to increased appetite, affecting the quantity and quality of the diet (increased sweet and high fat food intake), and body weight gain. Thus, exposure to stress can modify eating behavior. These stress-induced alterations in food intake and energy balance then interacting with emotional state and health cardiovascular, Figure 1 (Epel et al., 2001; Groesz et al., 2012). Though a relationship between nutritional changes and emotional state and brain health is already known to exist, the exact nature of this relationship has not been established. Research over the last decade has focused on bidirectional communication between gut and brain, termed the gut-brain axis. Dysbiosis and inflammation of the gut have been linked to several mental illnesses including anxiety and depression, although the molecular pathways involved have not yet been elucidated (Clapp et al., 2017).

The aim of this mini-review is to summarize the findings in the literature on how stress influences the molecular pathways affecting behavior, dietary patterns (DP) and their impact on health, and the synergistic effects of this cycle.

Figure 1 The potential biological mechanisms that connect the observed relationships between DP, stress and mental health could be modulated by the immune and endocrine systems, brain plasticity and the microbiome-gut-brain axis, being key targets for nutritional therapy in future research.

## Methods

The authors conducted a literature search of available sources describing issues relating to stress, DP and behavior. Research studies were selected based on research topics found in globally acknowledged databases such as Web of Science, PubMed, Springer, and Scopus, from 2010 up to the present time and classified according to their relevancy. The information provided in the selected studies was carefully evaluated and is described and discussed in the following sections.

## Nutrition and Cardiovascular Disease

Diet is one of the most studied factors in the pathogenesis of CVD since it affects diverse cardiometabolic risk factors such as obesity, lipid blood profile, blood pressure, glucose-insulin homeostasis, endothelial health, adipocyte metabolism, cardiac function, metabolic expenditure, weight regulation, visceral adiposity, and the microbiome (Mozaffarian, 2016).

Dietary patterns refers to the habitual combinations of foods and meals consumed on a daily basis (Hu, 2002). Several DP have been related to beneficial cardiovascular effects. For example, the Mediterranean DP -characterized by moderate energy intake, high consumption of olive oil, fish, legumes, nuts, fruits, and vegetables; fewer red meats and processed (sodium-preserved) meats; and a regular and moderate consumption of red wine- is associated with low CVD incidence and mortality (Shen et al., 2015; Sanches Machado d’Almeida et al., 2018). This DP and others such as Prudent, DASH or Healthy DP are high in fiber, vitamins, antioxidants, minerals, phenolics, and unsaturated fats, and low in glycemic index, glycemic load, salt, and *trans* fat (Esmaillzadeh et al., 2007; Mozaffarian et al., 2011). The Western DP on the other hand, characterized by a high intake of red meats, fat dairy products, refined grains and sugars, has been positively correlated with higher concentrations of markers of endothelial dysfunction, the first step in CVD: fasting insulin, C peptide, leptin, C reactive protein, homocysteine, tissue plasminogen activator antigen, interleukin 6 (IL-6), E-selectin, intercellular adhesion molecule 1 (sICAM-1) and vascular cells of molecular adhesion 1 (sVCAM-1) (Defagó et al., 2014; Rodríguez-Monforte et al., 2015; Marchiori et al., 2017).

In the long term, not only the quality but also the quantity of nutrients consumed can influence the neural circuits that regulate motivation, emotion and mood. Evidence can be found in literature about the relationship between saturated, *trans* fat intake and the risk of mental disorders (Barnard et al., 2014). Chronic exposure to a high-fat diet may affect the underlying neurobiological pathways of emotion and reward via its action on energy metabolism, endocrine function and immunity. Saturated and *trans* fatty acids favor central fat deposition and have been related with cardiometabolic and neurological diseases (Micha and Mozaffarian, 2008). The omega-3 polyunsaturated fatty acids (PUFAs) participate in modulation inflammation and immunospecific response, cell growth and tissue repair. Omega-3 plays a role in neuronal membrane fluidity and receptor function and lower levels of these PUFAs have been associated with common mental disorders such as depression and generalized anxiety, also with accelerated neurodegeneration (Grant and Guest, 2016).

Acute stress exposure (short term exposure) may shut down appetite by corticotropin-releasing hormone action and epinephrine liberation. However, if the stressor agent persists, the increased cortisol increases appetite and the motivation to eat (Razzoli et al., 2017). Experimental studies have demonstrated how chronic stress exposure increases susceptibility to diet-induced obesity, with induced spontaneous binging and hyperphagia and a preference for highly-palatable food, rich in calories, *trans* fat, salt and sugar (Packard et al., 2014; Ulrich-Lai et al., 2015). Research on human behavior shows a strong link between exposure to stress and binge eating disorder, often associated with development the overweight and obesity. Stress-induced non-nutritive food selection, is often referred to as eating “comfort food” or highly palatable food (Leigh et al., 2018).

## Nutrition, Microbiome and Cardiovascular Disease

The human gut microbiome contains 10^14^ resident microorganisms, among which bacteria are the most well-studied group, predominated by gram positive *Firmicutes* and gram negative *Bacteroidetes* (Cresci and Bawden, 2015). The collective genome of the microbiome contains millions of genes compared to the approximately 25,000 genes of the human genome and thus contributes to a wide range of biochemical and metabolic functions, such as nutrient acquisition, the harvesting of energy and large numbers of host metabolic pathways.

Inflammatory related diseases has been related to intestinal microbiome composition (bowel and skin diseases, autoimmune arthritis, type 2 diabetes, and atherosclerosis among others) (Buford, 2017). Specifically, an abnormal change in gut flora has been linked to a range of CVD risk factors, including obesity and diabetes. The first analyses on human intestinal microbiota reported a lower amount of *Bacteroidetes* than *Firmicutes* in obese individuals, however, these findings have not been consistently demonstrated in all subsequent metagenomic studies on obesity in humans (Ley, 2010). A lower abundance of butyrate-producing bacteria, in particular *Faecalibacterium prausnitzii*, a major member of the *Firmicutes phylum*, in individuals with obesity-related metabolic disturbances and diabetes has been observed. Furthermore, the presence of this bacterium is inversely correlated with the intake of dietary fat. Other studies have identified increased abundance of *Bifidobacteria* species in healthy individuals compared with individuals with obesity and diabetes (Martín et al., 2017). Dysbiosis has also been linked to CVD and metabolic diseases such as a lower ratio of *Bacteroidetes* to *Firmicutes* in obesity and hypertension, or increased *Collinsella* in atherosclerosis (Brahe et al., 2016).

Several DP have been studied for their ability to modulate the intestinal microbiota. In general terms, a Western DP led to a marked decrease in numbers of total bacteria and beneficial *Bifidobacterium* and *Eubacterium* species, whereas Mediterranean and Prudent DP have been linked to increases in *Lactobacillus, Bifidobacterium*, and *Prevotella*, and decreases in *Clostridium* (Koloverou et al., 2016; Singh et al., 2017*)*. However, dietary manipulation to modify gut microbiome is still at the incipient stage It has been detected that dietary fiber including prebiotics has a beneficial effect on gut microbiota and host health, improving insulin sensitivity, low-grade chronic inflammation, and lipid metabolism (Ahmadmehrabi and Tang, 2017). Prebiotics are non-digestible dietary compounds that stimulate growth or activity of autochthonous microorganisms, resulting in health benefits. They are basically oligo- or polysaccharides of fructose or galactose and also lactulose and lactitol; they promote the development of *bifidobacteria*, which are capable of degrading various complex carbohydrates. Many *Firmicutes* and in particular *Bacteroides* are also able to carry out this function (Suárez, 2013). Prebiotics are naturally present in foods such as wheat, garlic, onion, asparagus, leek, beet, chicory root, and artichoke, among others; they can also be added to products such as milk, yogurt and breakfast cereals. In this regard, adherence to a DP rich in fruits, vegetables and whole grains may modulate the gut microbiome and thus favorably impact on human health.

## Gut Brain Axis and Stress

The mechanisms underlying microbiota-gut-brain axis communication involves neuro-immune-endocrine mediators. This interconnected network includes the central nervous system (CNS), the autonomic nervous system, the enteric nervous system and the HPA (Farzi et al., 2018).

In an organism exposed to stress, the hypothalamus stimulates the pituitary by releasing corticotropin-releasing factor (CRF). In the anterior pituitary, CRF causes the release of the adrenocorticotropic hormone (ACTH), which in turn stimulates the adrenals to cause an increased rate of synthesis and release of cortisol (cortisol in humans and corticosterone in rodents) (Foster et al., 2017). Cortisol serves to maintain homeostasis during normal states of activity (regulating cellular function and metabolism) and during periods of stress (Christiansen et al., 2007).

Chronically elevated cortisol levels can disturb brain function, affecting cognition, emotion and motivation, short and long-term memory; sustained HPA axis activation also impairs the immune system response, as has been demonstrated particularly in early life stress (Shirtcliff et al., 2009). Cortisol exerts it’s effects trough the interaction with two corticoid receptors, high affinity mineralocorticoid receptors (MR) and low affinity glucocorticoid receptors (GR). A persistently activated HPA axis will eventually lead to a compensatory downregulation of the expression of GR signaling through epigenetic modifications (Weaver et al., 2004). In addition, glucocorticoids increase intestinal permeability, and negatively affects the gut microbiota composition.

Stress-related changes could be also mediated by neuroendocrine hormones, such as norepinephrine (NE) and dopamine (DA); it has long been known that these catecholamines can increase the growth rate of Gram-negative bacteria (Lyte and Ernst, 1992).

In turn, changes in gut microbiome composition has been already related to cognitive disorders and mental illness. There is strong evidence linking major depression and microbiome, and preclinical evidence related to anxiety disorders (Foster and McVey Neufeld, 2013; Malan-Muller et al., 2017).

## Linking Microbiome and Neuronal Signaling: Mechanistic Evidence

Further research is required to elucidate the exact mechanisms and mediators involved in brain-microbiota communication. A central issue that remains unresolved is which mediators the microbiota uses to communicate with the brain and how this network ultimately influences behavior and health.

There are three major systems along with their mediators playing a role in inter-talk between the microbiota and the brain: neuronal communication, endocrine signaling mediators and the immune system mediators. Together, these systems create an integrated molecular network that impact both in gut and brain function.

The gut communicates with the brain through a systemic, central route. The vagus nerve sends information to the brain, where it is processed in the nucleus tractus solitarius; this nucleus then projects to the parabrachial nucleus, which in turns projects to the prefrontal cortex (PFC) and amygdala. These latter are key loci that control anxiety and fear responses. It has been reported that both regions display irregularities in germ free animals (GF), including hypermyelination in the PFC.

Previous papers have shown that the *N*-methyl-D-aspartate (NMDA) receptor subunit (NMDArec2B) has reduced expression in the amygdala of GF animals (Neufeld et al., 2011). Furthermore, it has been demonstrated that feeding prebiotics to these animals elevates their levels of brain-derived neurotrophic factor (BDNF), NMDA receptor subunits, and D-serine in the brain. Moreover, GABA receptor and serotonin 1A receptor are upregulated in the amygdala and hippocampus, respectively, upon supplementing GF animals with a Lactobacillus strain, ultimately regulating behavior in mice (Bravo et al., 2011).

The biogenic amine Serotonin [5-hydroxytryptamine (5-HT)] exerts its function in the brain and in the ENS. Most of 5-HT is synthetized by gut mucosal enterochromaffin cells and ENS neurons. Peripherally, 5-HT is involved in gastrointestinal function, smooth muscle contraction and relaxation, and in pain perception. In the brain it is involved in regulating mood and cognition (Yano et al., 2015). Serotonin synthesis depends on the availability of tryptophan, an essential amino acid that must be supplied in the diet, and microbiota plays a central role in the regulation and synthesis of this amine.

Together these findings reinforce the concept that DP can have either a positive or a negative impact on the CNS by regulating critical neurotransmitters implicated in psychiatric disorders, such as depression (Wallace and Milev, 2017; Zalar et al., 2018).

Finally, the immune system plays an important intermediary role between the brain and the gut. Gut-associated lymphoid tissue is the main component of mucosal-associated lymphoid tissue and corresponds to almost 70% of the entire immune system (Vighi et al., 2008). Dysbiosis of the gut microbiota is linked to abnormal immune responses, which are accompanied by irregular cytokine synthesis and release. It has been reported that TNFα and IFNγ production capacity appears to be influenced by the microbiome, whereas cytokines such as IL-1β, IL-6, and Th17-derived IL-17, and IL-22 display fewer, but more specific, associations with the gut microbiota (Schirmer et al., 2016).

Table 1 Mechanistic pathways, mediators and main microbiome modifications associated with stress related psychological disorders. Molecular signaling pathways linking the microbiome with stress-related health outcomes.

**TABLE 1:** Molecular signaling pathways linking the microbiome with stress-related health outcomes.

**Pathway**	**Mediator**	**Microbiome**	**Health outcome**	**References**
Neural pathways	Serotonin	GF vs. specific pathogen-free animals (SPF)	Anxiety-like behavior	Neufeld et al., 2011; Clarke et al., 2012
	GABA	*Bacteroides*, *Parabacteroides* and *Escherichia* species	Depression	Strandwitz et al., 2018
	miRNA	GF vs. GF colonized animals	Anxiety- and fear-related behavior	Hoban et al., 2017
	c-FOS	*Campylobacter jejuni*	Anxiety-like behavior	Lyte et al., 2006; Goehler et al., 2008
	BDNF	GF animals	Brain development	Diaz Heijtz et al., 2011
Endocrine pathways	Cortisol	*Lactobacillus helveticus, Bifidobacteria*	Anxiety-like behavior	Foster et al., 2017
Immune signaling	TNF-α	*Lactobacillus plantarum* PS128	Improved anxiety-like behavior	Liu et al., 2016
	IL-6	*Lactobacillus*	Inflammation, overweight, obesity.	Cooper et al., 2016
	IL-1β	*Akkermansia* spp. and *Blautia* spp.	Depression	Marc et al., 2014; Wong et al., 2016

Several studies related to preclinical models of bacterial infections, probiotics treatment and analysis of germ-free (GF) animals suggest that the microbiota can influence the CNS and consequently behavior. Stress-related disorders such as anxiety and depression are among the main psychiatric conditions associated with microbiome changes (Desbonnet et al., 2010, 2015; Bravo et al., 2011).

## Conclusion

The treatment of multidimensional diseases such as CVD require multidimensional approaches. Nutritional psychiatry is a growing field of research seeking to provide clinically relevant interventions for multifactorial diseases. This emerging line of inquiry has assembled data on biological pathways such as gastrointestinal microbiota and inflammation, on DP and on how these are modulated by the environment. It has become clear that this intertwined network of environmental factors (such as stress and DP) interacting with the above-mentioned pathways combines to modulate behavior.

Some trending foods and (controversial) diets have brought lasting changes to traditional diets, however, in most cases neither these nor dietary supplements have been subjected to adequate clinical trials to test their safety and efficacy. A better understanding of the interplay between diet, stress, neuronal signaling, phenotypes, and the microbiome will provide important insights into the utility of scientific evidence-based nutrition. Future studies are imperative to proactively avoid cardiovascular events in patient population.

## Author Contributions

All authors equally contributed to the drafting, analyses the final version of the manuscript, and read and approved the final version of the manuscript.

## Conflict of Interest

The authors declare that the research was conducted in the absence of any commercial or financial relationships that could be construed as a potential conflict of interest.
